# The Effects of Leucine, Zinc, and Chromium Supplements on Inflammatory Events of the Respiratory System in Type 2 Diabetic Rats

**DOI:** 10.1371/journal.pone.0133374

**Published:** 2015-07-17

**Authors:** Saeed Kolahian, Hassan Sadri, Amir Ali Shahbazfar, Morvarid Amani, Anis Mazadeh, Mehdi Mirani

**Affiliations:** 1 Department of Basic Sciences, Faculty of Veterinary Medicine, University of Tabriz, Tabriz, Iran; 2 Institute of Animal Science, Physiology and Hygiene Unit, University of Bonn, Bonn, Germany; 3 Department of Clinical Sciences, Faculty of Veterinary Medicine, University of Tabriz, Tabriz, Iran; 4 Department of Pathobiology, Faculty of Veterinary Medicine, University of Tabriz, Tabriz, Iran; 5 Faculty of Veterinary Medicine, University of Tabriz, Tabriz, Iran; University of Tübingen, GERMANY

## Abstract

Diabetes mellitus is a major cause of serious micro- and macrovascular diseases that affect nearly every system in the body, including the respiratory system. Non-enzymatic protein glycation due to hyperglycaemic stress has fundamental implications due to the large capillary network and amount of connective tissue in the lung. The current study was designed to determine whether leucine, zinc, and chromium supplementations influence the function and histological structure of the respiratory tract in a rat model of type 2 diabetes. Seventy-seven rats were divided into eleven groups, consisting of 7 animals each. One group served as negative control and insulin and glibenclamide were used as positive control drugs. Thus, eight groups received the nutritional supplements alone or in combination with each other. Nutritional supplements and glibenclamide were added to the drinking water and neutral protamine Hagedorn insulin was subcutaneously injected during the 4 weeks of treatment period. The induction of type 2 diabetes in the rats caused an infiltration of mononuclear cells and edema in the submucosa of the trachea and lung, severe fibrosis around the vessels and airways, and perivascular and peribronchial infiltration of inflammatory cells and fibrin. In the diabetic group, the total inflammation score and Reid index significantly increased. Diabetes induction significantly reduced the total antioxidant status and elevated the lipid peroxidation products in the serum, lung lavage and lung tissue of the diabetic animals. Treatment with nutritional supplements significantly decreased the histopathological changes and inflammatory indices in the diabetic animals. Supplementation of diabetic rats with leucine, zinc, and chromium, alone and in combination, significantly increased the total antioxidant status and lipid peroxidation level in the diabetic animals. The nutritional supplements improved the enzymatic antioxidant activity of catalase, glutathione peroxidase, myeloperoxidase, and superoxide dismutase in the diabetic rats. The present results demonstrate beneficial effects and amelioration of inflammation in the respiratory system of type 2 diabetic rats by leucine, zinc, and chromium supplements, probably due to their hypoglycaemic and antioxidant properties. Using safe and effective nutritional supplements, such as leucine, chromium and zinc, to replace proven conventional medical treatments may help to control diabetes and/or its complications.

## Introduction

There are limited and contradictory data on the association between diabetes and pulmonary function. Type 2 diabetes mellitus is the most common endocrine disorder in the world. This condition is characterized by decreases in insulin secretion, defects in glucose uptake in skeletal muscle and adipose tissue and increases in glucose production in the liver [1,2]. The latter two abnormalities are primarily due to insulin resistance [3]. High dietary fat intake is associated with insulin resistance, and this represents a major risk factor for type 2 diabetes [4]. Diabetes is known to affect various organ systems, including the kidneys, retina, nerves, and cardiovascular system [5]. Recently, in the context of ongoing research into the pulmonary safety of inhaled insulin [6–8], the effects of diabetes on pulmonary function has also become a subject of interest [9–11]. Considering its large vascular network and richness in collagen and elastin, the pulmonary system is susceptible to undergoing microvascular damage and nonenzymatic glycation in diabetes. Many authors have investigated the pulmonary function and diffusion capacity of patients with diabetes, but their findings have been inconsistent [12]. In the present environment, the use of nutritional supplements has dramatically increased throughout the world, and major pharmaceutical companies are currently conducting extensive research on natural materials for their potential medicinal values. From both in vitro and in vivo studies in humans and laboratory animals, it has become evident that leucine (Leu) functions as a strong insulin secretagogue [13–15]. Induction of insulin secretion by leucine is mediated by its oxidative decarboxylation, by allosteric activation of glutamate dehydrogenase and by increasing the oxidation of glutamate [13]. Zinc (Zn), the second most abundant trace element in the body, plays a role in systemic glycemic control through its effects on insulin biosynthesis within pancreatic β-cells and probably by modulating the insulinaemic effects on target tissues [16]. On the other hand, chromium (Cr) is biologically active as a component of the oligopeptide chromodulin (also known as low-molecular-weight Cr-binding substance), which is part of an insulin-signalling pathway [17,18]. It has been shown that chromodulin stimulates both the tyrosine kinase activity of the insulin-activated insulin receptor and the membrane phosphotyrosine phosphatase in insulin-sensitive cells [17,18].

Using nutritional supplements, such as Leu, Zn, and Cr, that are closely associated with insulin metabolism may provide exciting new information and open up new possibilities for the prevention or control of insulin resistance and its associated disorders, including respiratory system abnormalities. However, to our knowledge, the question of whether supplementing the diet with Leu, Zn, and Cr affects the respiratory system in patients with type 2 diabetes has not been addressed. The current study was designed to determine whether Leu, Zn, and Cr supplementation influences the respiratory system in a rat model of type 2 diabetes.

## Materials and Methods

### Experimental groups

Seventy-seven adult male Wistar rats that were 8 weeks old (mean weight 150–200 g) were used in this study. The rats in each group were housed separately in single cages placed in a room with a 12:12-hour light:dark cycle and an ambient temperature of 22 ± 2°C. They were fed a commercially available food pellet diet (normal diet) and water *ad libitum* during the acclimation period (10 days). The blood glucose concentrations for all animals were determined with a blood glucose monitor (Glucose monitor, Canada) in samples obtained from the tail vein. All animals showed glucose levels of 80 ± 5 mg/dl. Afterwards, the rats were randomly categorized into eleven groups of 7 animals each: 1) control (**CTR**); 2) diabetic (**T2D**); 3) T2D + neutral protamine Hagedorn (NPH) insulin (**INS**); 4) T2D + glibenclamide (**GLC**); 5) T2D + leucine (**Leu**); 6) T2D + zinc (**Zn**); 7) T2D + chromium (**Cr**); 8) T2D + Leu + Zn (**Leu-Zn**); 9) T2D + Leu + Cr (**Leu-Cr**); 10) T2D + Zn + Cr (**Zn-Cr**); 11) T2D + Leu + Zn + Cr (**Leu-Zn-Cr**).

All of the procedures in this study were performed in accordance with the guidelines for the Care and Use of Laboratory Animals as adopted by the Ethics Committee of the Faculty of Veterinary Medicine of University of Tabriz, Iran (Permit Number: A-12-30511).

### Diabetes type 2 induction

The rats in the non-diabetic control group (CTR) were fed a normal chow diet consisting (as a percentage of total kcal) of 12% fat, 60% carbohydrate, and 28% protein (Javaneh Khorasan Co., Mashhad, Iran), whereas the diabetic groups were fed a high-fat diet (HFD) consisting of 40% fat, 42% carbohydrate, and 18% protein (Javaneh Khorasan Co., Mashhad, Iran). After 2 weeks of dietary manipulation and an overnight fast, the rats on the HFD were intraperitoneally injected with a low dose of streptozotocin (STZ) (35 mg/kg; Sigma-Aldrich, Inc., St. Louis, Mo., USA) [19]. The animals had free access to food and water after the STZ injection, and both the STZ-injected and CTR animals continued to receive their original diets for the entire course of the study.

### Diabetes confirmation

Three days after the STZ injection and an overnight fast, the presence of diabetes was verified by blood glucose concentrations above 250 mg/dl, as determined using a blood glucose monitor (Glucose monitor, Canada) in samples obtained from the tail veins.

### Treatment with nutritional supplements

After confirmation of the induction of diabetes, Leu (AllMax, USA) 15 g/L [13], Zn (Zinc sulphate, Alhavi, Iran) 10 mg/L [20] or Cr (Picolinate chromium, Swanson, USA) 5 mg/L [21] were added to the drinking water of the diabetic rats for 4 weeks. In addition, a subset of the diabetic rats received NPH insulin (an intermediate-acting insulin; Isophane Lansulin, Exir, Iran) in a dose of 2 U/day subcutaneously, or glibenclamide (glyburide, Iran Najo, Iran) at 20 mg/L for 4 weeks beginning on the same day as the addition of the nutritional supplements.

### Haematological analysis

The animals in all groups were killed by exsanguination at the end of each experimental protocol, and blood samples were collected following heart sectioning. The blood samples were transported and stored in the laboratory where the analyses of the haematological parameters were performed. The packed cell volume (PCV) was determined by the microhaematocrit method. The serum total protein and fibrinogen levels were investigated using Biuret and sedimentation methods, respectively. The blood haemoglobin was assessed based on the cyanohaemoglobin method. The red blood cells (RBCs), white blood cells (WBCs) and differential WBC counts (lymphocytes, neutrophils, monocytes, eosinophils and basophils) were assessed using an improved Neubauer haemocytometer.

### Measurement of inflammatory cell infiltration

The lungs were lavaged with three separate 2-ml aliquots of phosphate-buffered saline. The recovered bronchoalveolar lavage fluid was centrifuged (400 *g*, 10 min), and the pellet was resuspended in 200 μl of phosphate buffered saline. The cells were counted on a haemocytometer. The differential cell counts were performed on Cytospin preparations stained with Giemsa stain (Merck, Darmstadt, Germany) using light microscopy. A total of 200 cells was counted and classified as neutrophils, eosinophils, or mononuclear cells based on normal morphological criteria.

### Histopathology and scoring

The animals in all of the groups were killed by exsanguination at the end of experimental protocol (4 weeks after diabetes confirmation). The left diaphragmatic lobe of the lung and the trachea were removed and placed into 10% neutral phosphate-buffered formalin. After complete fixation, the tissues were processed using a tissue processor to pass them through increasing concentrations of ethanol followed by xylol for tissue dehydration and clearing. Paraffin blocks were prepared from the tissue samples. The specimens were cut into 4-μm slices and stained with haematoxylin and eosin (H&E stain). The tissues were examined using a light microscope. Perivascular and peribronchial inflammation were evaluated by counting the layers of inflammatory cells formed around the vessels and airways. At least 20 airways and 20 vessels were studied in each animal, and the average of the layers was calculated. Tracheal inflammation and glandular hyperplasia and hypertrophy were measured by calculating the Reid index. The Reid index is a parameter that is measured as follows: submucosal glandular layer thickness/tracheal wall thickness from the basement membrane to tracheal cartilage. All of the qualitative histological changes were recorded.

### Biochemical analysis of the samples

Blood was collected into test tubes by cardiac puncture, allowed to clot and centrifuged at 1000 × *g* to obtain the serum. The lung and tracheal extracts were prepared by snap freezing the samples in liquid nitrogen, homogenizing with a hand homogenizer and suspending the homogenates in cold 0.1 M phosphate-buffered saline (pH 7.2). The suspensions were centrifuged for 10 min at 4,000 × *g*.

### Lipid peroxidation assay in serum

The malondialdehyde (MDA) levels in the serum were determined using the thiobarbituric acid test [22], also known as the TBARS assay (thiobarbituric acid reactive substances assay). To precipitate the serum proteins, 2.5 mL of 20% (w/v) TCA was added to 0.5 mL of the sample, and the mixture was then centrifuged at 1,500 × *g* for 10 min. Then, 2.5 mL of 0.05 M sulphuric acid and 2 mL of 0.2% thiobarbituric acid (TBA) were added to the pellet, and this mixture was shaken and incubated for 30 min in a boiling water bath. Then, 4 mL n-butanol was added and the solution was centrifuged and cooled. Finally, the absorption of the supernatant was recorded at 532 nm using a UV/VIS spectrophotometer. A calibration curve was obtained using a range of concentrations of MDA as a standard to determine the concentration of TBA—MDA adducts in the samples, and the results were expressed as nmol/dL of serum [22].

### Tissue lipid peroxides

The total lipid peroxidation product, as indicated by MDA formation in the homogenate, was assayed using the TBA method [23]. Briefly, 2 ml of the TCA—TBA solution (0.67% TBA in 20% TCA) was added to 1 ml of the homogenate, mixed thoroughly and heated for 30 min in a boiling water bath. After cooling and centrifuging at 1,500 × *g* for 10 min, the absorbance of the supernatant was read at 535 nm in a spectrophotometer.

### Total antioxidant power assay

The ferric-reducing ability of plasma assay (FRAP), which measures the reduction of the ferric tripyridyltriazine [Fe(III)–TPTZ] complex to the ferrous tripyridyltriazine [Fe(II)–TPTZ] at low pH, was used to measure the total antioxidant capacity of the lavages, sera and tissue homogenates [24]. The Fe(II)–TPTZ complex gives a blue colour with an absorbance maximum at 593 nm. The results were expressed as equivalents of vitamin C, a potent antioxidant used for calibration purposes.

### Total protein assay

The total protein concentration was measured using the Lowry method based on the reactivity of the peptide nitrogen[s] with the copper [II] ions under alkaline conditions and the subsequent reduction of the Folin-Ciocalteu phosphomolybdic phosphotungstic acid to heteropolymolybdenum blue by the copper-catalysed oxidation of aromatic acids [25].

### Blood insulin level

The blood insulin levels were measured using a standard commercial ELISA kit (BioMark Technologies Inc., Canada).

### Blood and lung antioxidant enzyme activity levels

The blood and lung antioxidant activity levels were measured using standard methods [26–31].

### Measurement of catalase (CAT) activity

CAT was assayed colourimetrically at 620 nm and expressed as the moles of H_2_O_2_ consumed/min/mg protein as described by Sinha (1972). The reaction mixture contained phosphate buffer (0.01 M, pH 7.0), the tissue homogenate and 2 M H_2_O_2_. The reaction was stopped by the addition of the dichromate:acetic acid reagent (5% potassium dichromate and glacial acetic acid mixed in a ratio of 1: 3) [26,27].

### Measurement of superoxide dismutase (SOD) activity

SOD was measured based on inhibition of the formation of amino blue tetrazolium formazan in a nicotinamide adenine dinucleotide, phenazine methosulfate and nitroblue tetrazolium (NADH-PMS-NBT) system, according to method of Kakkar et al. [28]. One unit of enzyme activity was defined as 50% inhibition of NBT reduction [28].

### Measurement of glutathione peroxidase (GPx) activity

GPx activity was assessed by combining the samples with a mixture of 1 U/mL glutathione reductase and 2 mmol/L glutathione in 1 mL phosphate buffer. The mixtures were preincubated at 37°C for 30 minutes. Subsequently, NADPH and tert-butylhydroperoxide were added, and the change in absorbance at 340 nm was recorded to calculate GPx activity, as previously described [29,30].

### Measurement of myeloperoxidase (MPO) activity

MPO activity was determined using the o-dianisidine—hydrogen peroxide method. The change in absorbance was measured at 460 nm (ε 11 300/mol per cm) for 5 min. The results were expressed as units of MPO/mg protein, where 1 unit of MPO was defined as the quantity of enzyme that degraded 1 nmol of hydrogen peroxide/min at 25°C [31].

### Statistical analysis

In the histopathological data, the differences in the percentage of mucus cells and serous cells, Reid index, and perivascular and peribronchiolar infiltration of inflammatory cells were compared among the groups using one-way ANOVA. The rest of data were analysed using the MIXED procedure of SAS 9.2. The treatment was considered to be the fixed effect, and the rat was considered to be the random effect. The Tukey correction method was used for correction of the multiple comparisons. The data were expressed as the means ± SEM. The results are presented in the tables as the least squares means and standard errors of the means. The threshold of significance was set at *P*<0.05.

## Results

### Histopathological changes in the lungs and trachea

#### Ameliorative effects of nutritional supplements on inflammatory changes in the lungs and trachea of diabetic rats

**Fig 1:**
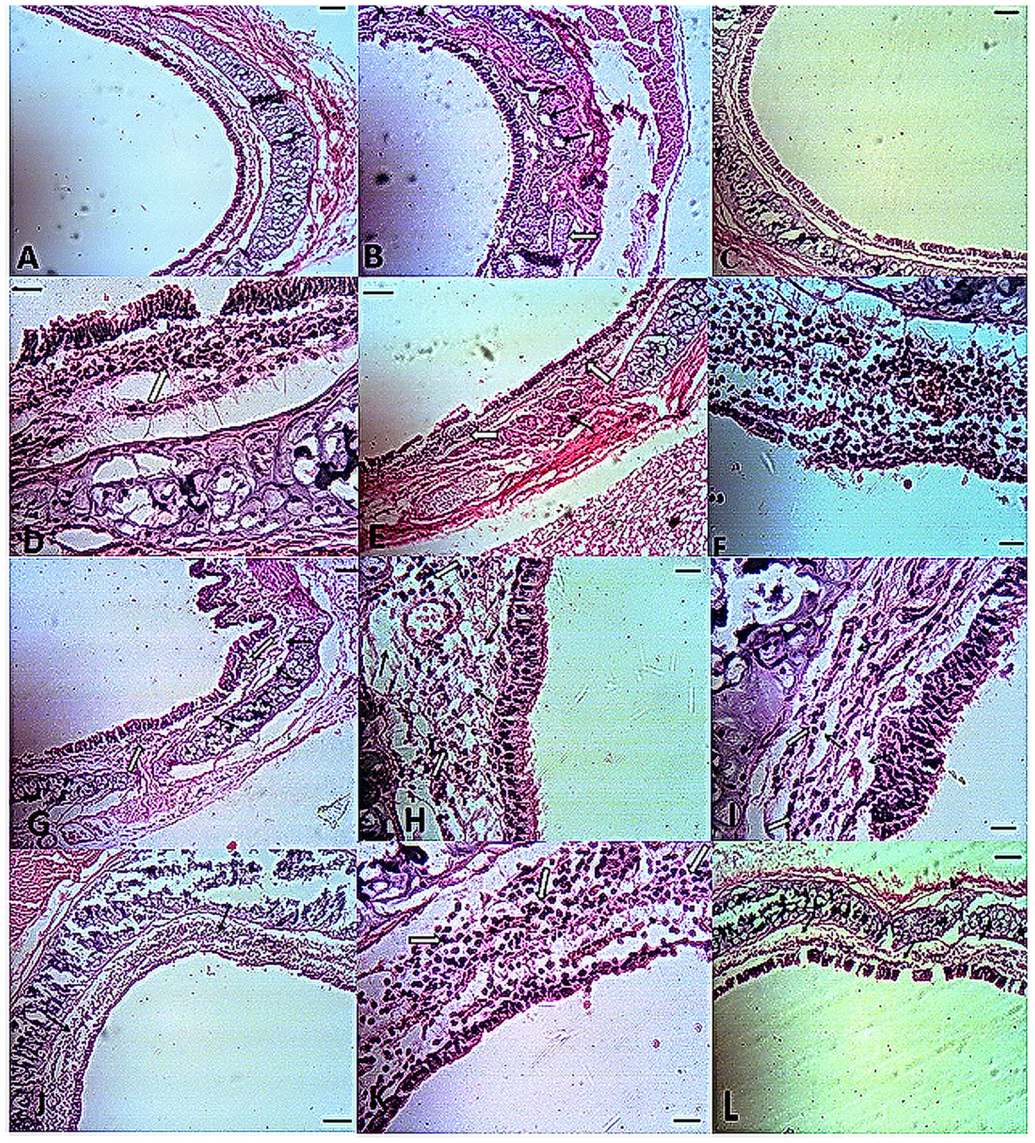
Histopathological findings in the tracheas of rats in all of the experimental groups. A. Control (CTR): normal architecture of trachea Bar: 125 micrometres; B. Diabetic group (T2D): sub-mucosal gland hyperplasia. Although most of the glands are serous glands (black arrows), one mucus secretory unit is observable (white arrow) Bar: 50 micrometres; C. Insulin-treated group (INS): the trachea is approximately normal. Bar: 125 micrometres; D. Glibenclamide-treated group (GLC): inflammatory cells are obvious in the lamina propria and submucosa (white arrows). Bar: 12.5 micrometres; E. Leucine-treated group (Leu): inflammatory mononuclear cells are present in the submucosa (white arrows). Hypertrophic and hyperplastic glands are present (black arrow) Bar: 50 micrometres; F. Zinc-treated group (Zn): severe inflammation in the lamina propria and submucosa. Bar: 12.5 micrometres; G. Chromium-treated group (Cr): edema (black arrows) and inflammation (white arrows) in the lamina propria and submucosa. Bar: 50 micrometres; H. Zinc and Cr-treated group (Zn-Cr): edema (black arrows) and slight inflammation (white arrows) in the lamina propria and submucosa. Bar: 12.5 micrometres; I. Leu plus Zn-treated group (Leu-Zn): edema (black arrows) and slight inflammation (white arrows) in the lamina propria and submucosa. Bar: 12.5 micrometres; J. Leu plus Cr-treated group (Leu-Cr): inflammation in the submucosa (arrows) Bar: 50 micrometres; K. Zinc plus Cr-treated group (Zn-Cr): the presence of different types of inflammatory cells in the lamina propria and submucosa (arrows) Bar: 12.5 micrometre; L. Leucine plus zinc plus chromium-treated group (Leu-Zn-Cr): slight edema in the submucosa (arrows) Bar: 50 micrometres.

**Fig 2:**
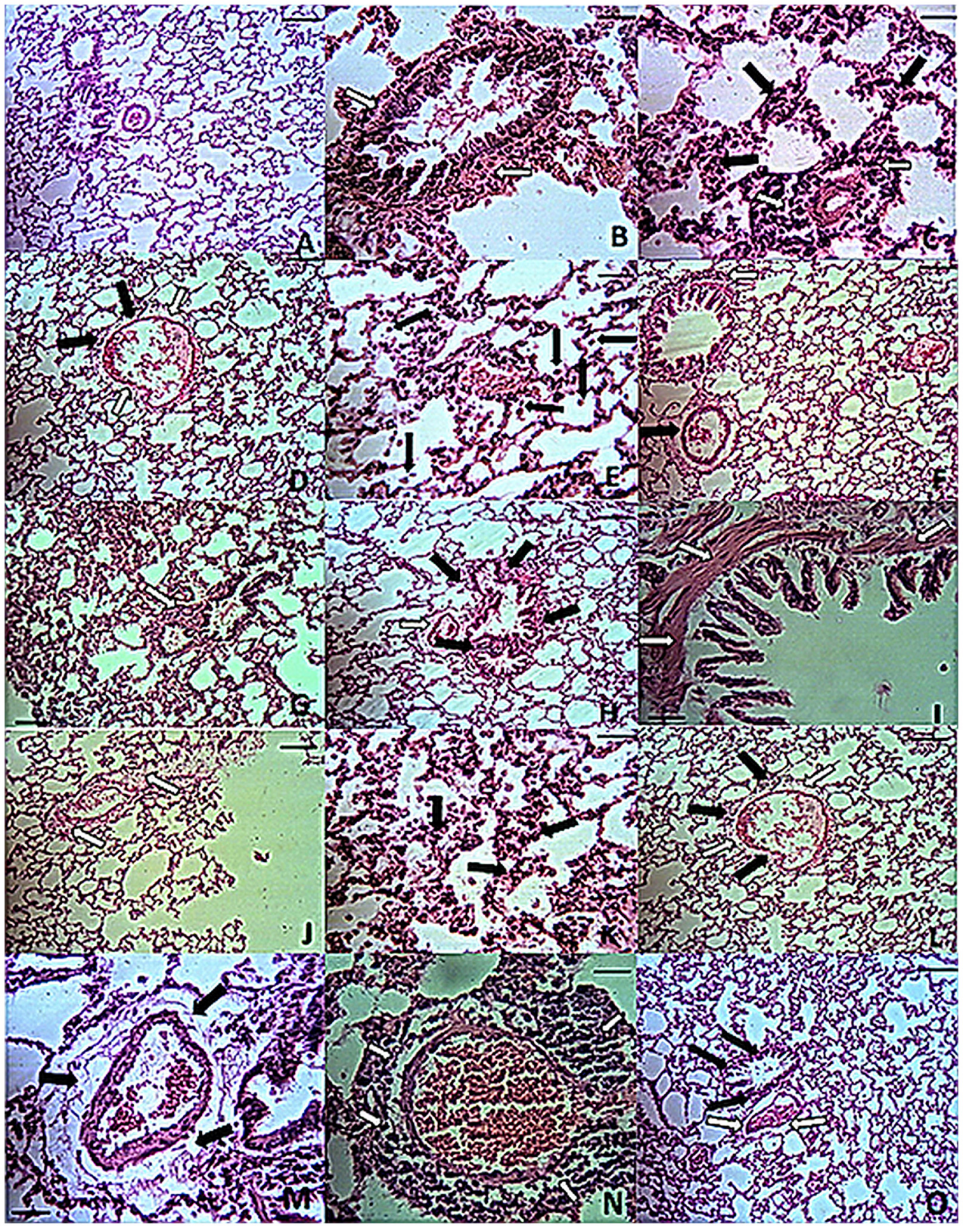
Histopathological findings in the lungs of rat in all of the experimental groups. Lung parenchyma. A. Control (CTR): lung normal parenchyma. Bar: 75 micrometres; B. Diabetic group (T2D): hypertrophy and hyperplasia of the smooth muscles in a secondary bronchiole wall (arrows). Bar: 20 micrometres; C. Diabetic group: severe inflammation around a blood vessel (white arrows) and alveolar epithelialization (black arrows). Bar: 75 micrometres. D. Insulin-treated group: slight edema (white arrows) and scattered inflammatory cells (black arrows) around a blood vessel. The lesions are less pronounced than those of the diabetic group. The alveolar walls are normal with less epithelialization. Bar: 75 micrometres. E. Leucine-treated group (Leu): increased numbers of intra-alveolar macrophage compared with the control (arrows). Bar: 75 micrometres. F. Glibenclamide-treated group (GLC): inflammatory cell infiltration around a secondary bronchiole (white arrow), edema and fibrin accumulation and fibrosis around a vessel (black arrow). Bar: 75 micrometres. G. Leu-treated group: severe inflammation around a secondary bronchiole (white arrow) and epithelialization in the alveolar walls. Bar: 75 micrometres. H. Zinc-treated group (Zn): Peribronchial inflammation (black arrows) and perivascular edema (white arrow). Bar: 75 micrometres. I. Zn-treated group: hypertrophy and hyperplasia of the smooth muscle in a secondary bronchiole wall (arrows) Bar: 20 micrometres. J. Cr-treated group: perivascular edema, fibrin accumulation and fibrosis (arrows). Bar: 75 micrometres. K. Chromium-treated group (Cr): alveolar epithelialization (arrows). Bar: 75 micrometre L. Zn plus Cr-treated group (Zn-Cr): perivascular edema and fibrin accumulation (white arrows) and scattered infiltration of inflammatory cells (black arrows). Bar: 75 micrometres. M. Leu plus Zn-treated group (Leu-Zn): perivascular edema and fibrin and collagen accumulation (arrows). Bar: 20 micrometres. N. Leu plus Cr-treated group (Leu-Cr): severe perivascular inflammation. Bar: 20 micrometres. O. Leu, Zn plus Cr-treated group (Leu-Zn-Cr): perivascular edema (white arrows) and slight infiltration of inflammatory cells (black arrows). The alveolar epithelialization is somewhat less than in the diabetic group. Bar: 75 micrometres.

Scattered infiltration of small numbers of inflammatory cells was observed in tracheal submucosa and lung parenchyma of the CTR group. There were no specific lesions in the respiratory system of this group. In the T2D group, infiltration of mononuclear cells and edema in the submucosa of trachea was observed. Hyperplasia and hypertrophy of the submucosal serous glands were obvious (Fig 1). In the lung, alveolar epithelialization (type II pneumocyte hyperplasia) increases the number of alveolar macrophages and leads to remodelling and fibrosis around the vessels and airways, which was more severe around vessels; perivascular infiltration of fibrin, inflammatory cells and edema; peribronchial infiltration of inflammatory cells; hyperaemia and scattered infiltration of mononuclear cells in the lung interstitium; and hypertrophy and hyperplasia of the bronchiolar muscle; and emphysema was also observed. Treatment with glibenclamide (GLC) decreased the inflammation and submucosal glandular hypertrophy and hyperplasia, perivascular and peribronchial edema and inflammation compared with the diabetic group. The extent of emphysema, increased alveolar macrophage numbers and alveolar epithelialization were less pronounced than in the T2D group, although they were still noticeable (Fig 2). Treatment with insulin (INS) decreased the inflammation of the lungs and trachea to a level comparable to all other treatment groups. The emphysema, edema and epithelialization were reduced compared to the other groups, and the alveolar macrophages were normal in number. There was no evidence of hypertrophy or hyperplasia of the bronchiolar muscle. Insulin decreased all of the pathological effects of diabetes, but the changes did not reach the control level. Leucine slightly decreased the numbers of alveolar macrophages. The numbers of alveolar macrophages and the inflammation in the lungs and trachea were reduced in the Zn and Cr groups compared to the T2D group. In the Zn-Cr, Leu-Zn and Leu-Cr groups, the numbers of alveolar macrophages and the inflammation in the lungs and trachea were less than T2D group. Co-treatment with Zn, Cr and Leu (Leu-Zn-Cr) markedly decreased the number of alveolar macrophages and the inflammation in the lungs and trachea compared with the T2D group (Figs 1 and 2).

#### Perivascular and peribronchial inflammation was reduced in the lungs of diabetic rats after treatment with nutritional supplements

**Fig 3:**
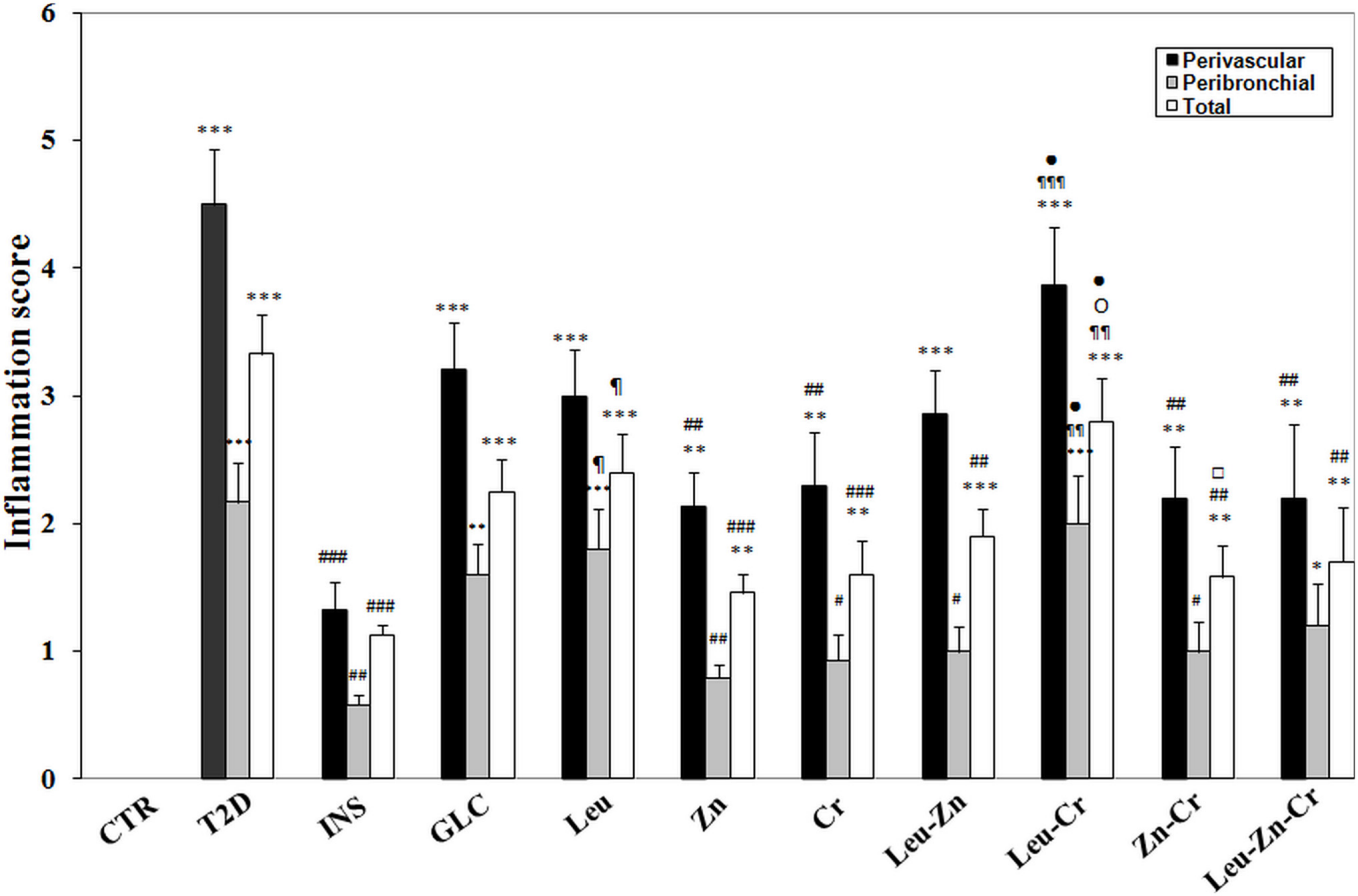
Perivascular, peribronchial inflammation and total inflammation scores in the rat lungs of all of the experimental groups. ***P<0.001, **P<0.01, *P<0.05 compared with the control. ###*P*<0.001, ##*P* <0.01, #*P*<0.05 compared with the diabetic group. ¶¶¶*P*<0.001, ¶¶*P*<0.01, ¶*P*<0.05 compared with the insulin-treated group. O*P*<0.05 compared with the chromium-treated group. •*P*<0.05 compared with the zinc-treated group. □*P*<0.05 compared with the leucine plus chromium group.

In the T2D group, perivascular cuffing (PVC), peribronchial cuffing (PBC) and the total inflammation score (the average of PVC and PBC) were significantly increased compared with the CTR group (*P*<0.001). Treatment with INS, but not GLC, significantly decreased the inflammation scores (*P*<0.001). Treatment with Cr and Zn significantly decreased inflammation compared with the T2D group (*P*<0.05 and *P*<0.01, respectively). Co-treatment with Leu and Zn effectively reduced the PBC (*P*<0.05) and the total inflammation score (*P*<0.01) compared with the T2D group. Co-treatment with Leu and Cr was not effective on the PVC, PBC and total inflammation scores, and these values were significantly higher than in the CTR (*P*<0.001) and INS groups (*P*<0.01). Chromium plus Zn, with or without Leu, was able to decrease these three inflammation scores compared with the T2D group (*P*<0.05; Fig 3).

#### The Reid index (RI) decreased in the lungs of the diabetic animals after using nutritional supplements

**Fig 4:**
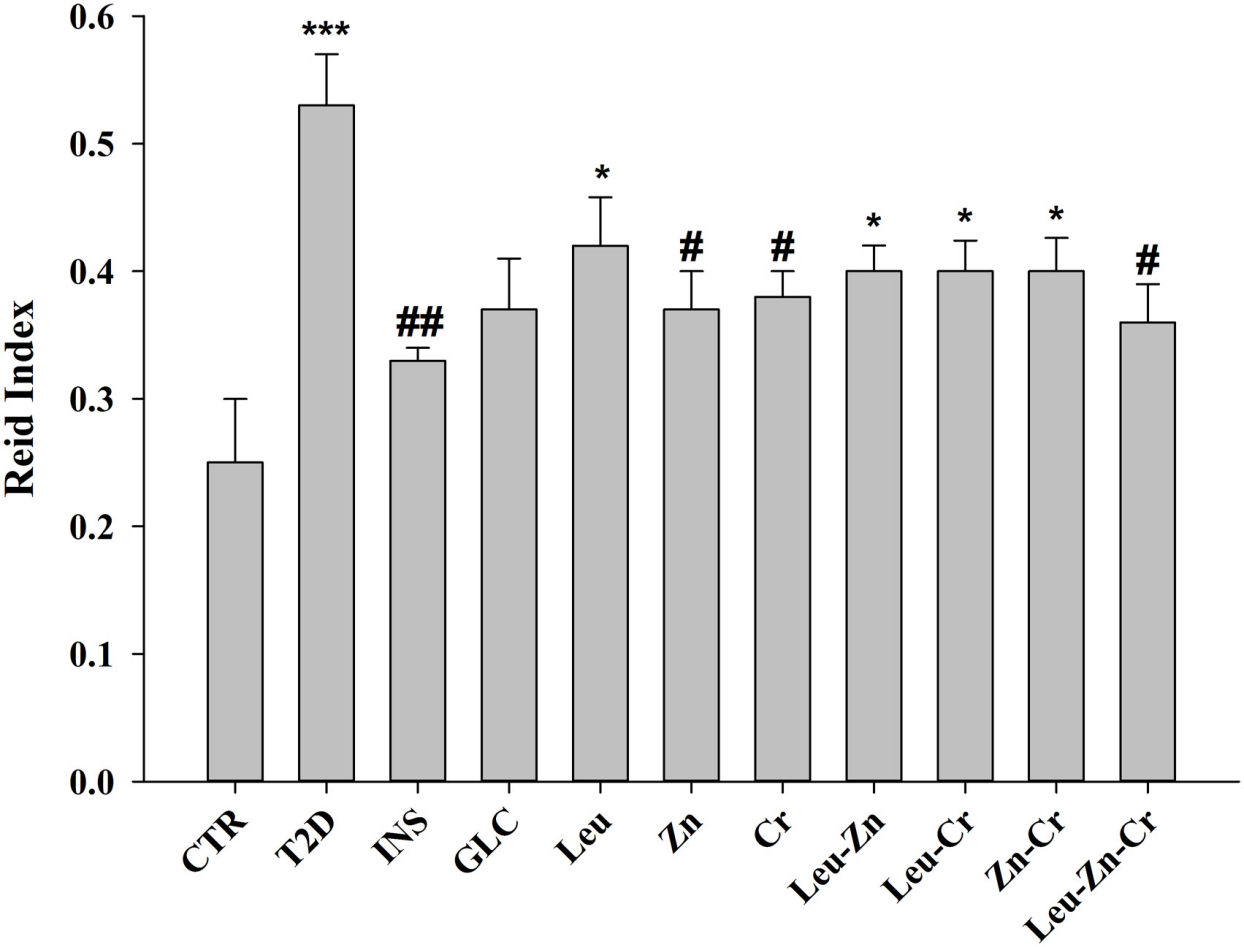
Reid index in the experimental groups. ***P<0.001, *P<0.05 compared with the control. ##*P*<0.01, #*P*<0.05 compared with the diabetic group. CTR = control; T2D = type 2 diabetes; INS = NPH insulin; GLC = glibenclamide; Leu = leucine; Zn = zinc; Cr = chromium.

The RI was significantly increased in the T2D group compared with the CTR group (*P*<0.001). The RI was significantly decreased in the Cr and Zn groups compared with the T2D group (*P*<0.05). Leucine alone and or in combination with Zn did not decrease the RI. Co-administration of Zn and Cr and co-administration of Leu and Cr were not effective on RI. Co-treatment with Zn, Cr and Leu significantly decreased the RI compared with the T2D group (*P*<0.05; Fig 4).

### Haematologic analysis

#### Blood parameter changes in diabetic rats were normalized by nutritional supplements

**Fig 5:**
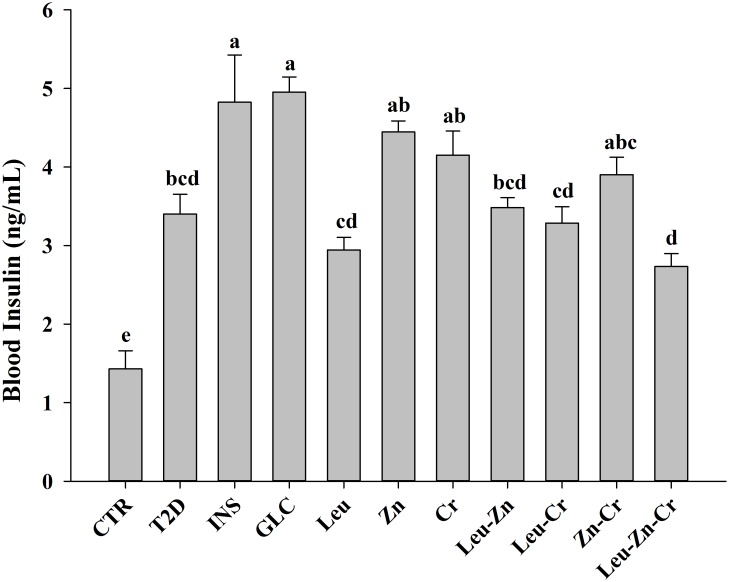
Blood insulin concentrations in the experimental groups. Different superscript letters (a-d) indicate significant differences among the groups (*P*<0.05). CTR = control; T2D = type 2 diabetes; INS = NPH insulin; GLC = glibenclamide; Leu = leucine; Zn = zinc; Cr = chromium.

Diabetes induction significantly increased the PCV and total blood protein, but these values were decreased in the Leu-Zn and Leu-Zn-Cr groups, respectively (Table 1). The serum fibrinogen was significantly increased in the T2D group compared with the CTR group. This increase was significantly attenuated in the Leu-Zn-Cr group (Table 1). The blood RBC number and Hb level were significantly increased in the T2D group compared with the CTR group. Administration of the nutritional supplements slightly reversed the effects of diabetes induction, but the effects were significant in only some of the treatment groups (Table 1). Diabetes induction significantly increased the insulin level in the T2D group compared with the CTR group. The Leu and Leu-Zn-Cr groups showed slight decreases in blood insulin concentrations, but the values were not as low as that of the CTR group (Fig 5). Furthermore, we did not find any significant differences in WBCs or the differential counts of the WBCs (lymphocytes, neutrophils, monocytes, eosinophils, and basophils) among the experimental groups (Table 1).

**Table 1:** Haematological parameters of the experimental groups. Data are least squares means and standard error of the mean.

	Treatments^2^												
Item^1^	CTR	T2D	INS	GLC	Leu	Zn	Cr	Leu-Zn	Leu-Cr	Zn-Cr	Leu-Zn-Cr	SEM	*P*-Value
PCV (%)	41.2^c^	48.5^a^	47.1^a^	48.5^a^	43.7^bc^	45.9^a^	44.9^abc^	44.9^abc^	44.6^abc^	44.9^abc^	43.1^bc^	0.96	<0.001
TP (g/dL)	41.2^c^	72.5^ab^	71.9^ab^	75.3^a^	65.1^bc^	67.6^abc^	66.7^abc^	64.9^bc^	66.3^abc^	65.6^abc^	67.4^abc^	2.21	0.002
FBG (mg/dL)	83.3^e^	325.0^abc^	425.0^a^	350.0^ab^	135.7^cde^	157.1^bcde^	278.6^abcd^	192.9^bcde^	228.6^abcde^	292.9^abcd^	114.3^de^	43.7	<0.001
RBC (10^6^/uL)	69.3^e^	84.5^ab^	79.7^abcd^	85.8^a^	71.0^de^	79.7^abc^	72.7^cde^	78.4^abcd^	75.9^bcde^	75.1^cde^	73.4^cde^	2.01	<0.001
Hb (g/dL)	138.3^d^	170.2^a^	162.3^ab^	170.5^a^	146.3^cd^	157.1^abc^	152.6b^cd^	152.9^bcd^	151.6^bcd^	151.7^bcd^	154.4^bc^	3.48	<0.001
WBC (cells/uL)	9983.3	10975.0	12117	12192.0	10391	11257.0	9320.0	12419.0	10493.0	9881.4	10451.0	954.6	0.38
Neutrophils (%)	17.0	18.8	27.2	26.5	18.7	19.3	18.1	23.0	21.6	22.6	21.6	2.23	0.06
Lymphocytes (%)	78.7	76.3	67	67.5	76.4	75.9	76.0	74.1	73.9	73.7	74.1	2.72	0.14
Monocytes (%)	2.50	2.17	3.33	4.00	2.14	2.43	2.43	1.57	1.57	1.71	2.43	0.65	0.41
Eosophils (%)	1.28	2.67	2.11	2.63	2.57	2.43	3.29	2.71	3.00	1.71	1.71	0.61	0.46
Basophils (%)	0.32	0.023	0.34	0.015	0.14	0.00	0.14	0.14	0.00	0.29	0.14	0.13	0.45

^1^PCV = packed cell volume; TP = total protein; FBG = fibrinogen; RBC = red blood cells; Hb = hemoglobin; WBC = white blood cells.

^2^CTR = control; T2D = type 2 diabetes; INS = NPH insulin; GLC = glibenclamide; Leu = leucine; Zn = zinc; Cr = chromium.

^a–e^Significant differences (*P*<0.05) among treatments are indicated by different letters.

#### Blood antioxidant enzyme activities improved using nutritional supplements in diabetic rats

**Fig 6:**
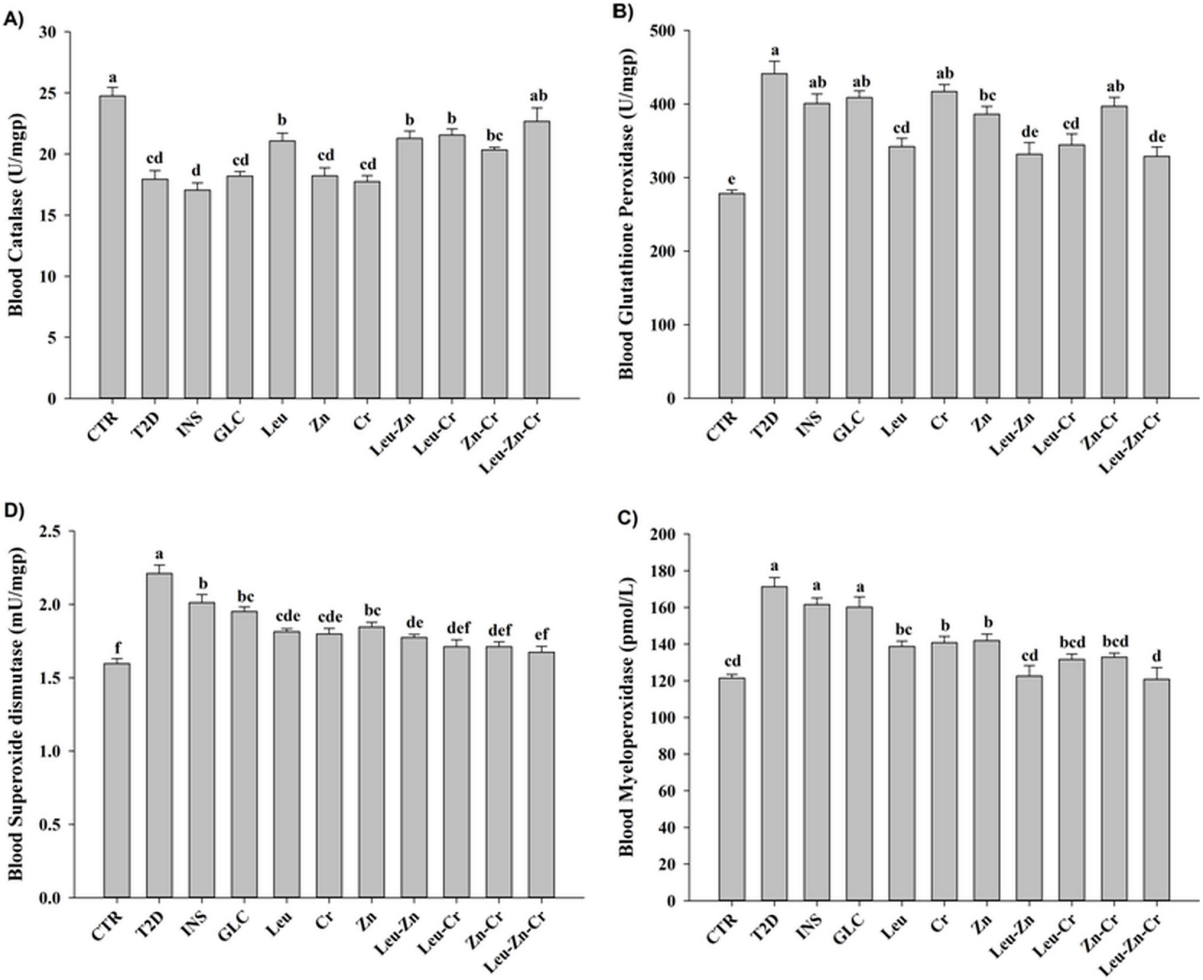
Blood antioxidant enzyme activities in the experimental groups. Blood catalase (A), glutathione peroxidase (B), myeloperoxidase (C) and superoxide dismutase (D) activities was measured in all of the experimental groups. Different superscript letters (a-f) indicate significant differences among the groups (*P*<0.05). CTR = control; T2D = type 2 diabetes; INS = NPH insulin; GLC = glibenclamide; Leu = leucine; Zn = zinc; Cr = chromium.

The level of catalase activity in the T2D animals was significantly lower than in the CTR animals (*P*<0.05). Treatment with INS or GLC had no significant effect on the catalase activity. Treatment with leu, Leu-Zn, Leu-Cr or Leu-Zn-Cr significantly increased the catalase activity in the T2D animals (*P*<0.05; Fig 6A). The glutathione peroxidase activity was significantly increased in the T2D group compared with the CTR group (*P*<0.05). The glutathione peroxidase activity was not affected by the INS or GLC treatments. Zn, Leu, leu-Zn, and leu-Zn-Cr significantly decreased the glutathione peroxidase activity in the diabetic animals (*P*<0.05; Fig 6B). The myeloperoxidase activity was significantly increased in the T2D group compared with the CTR group (*P*<0.05). Treatment with INS or GLC had no significant effect on the myeloperoxidase activity, but treatment with any of the nutritional supplements or their combinations significantly decreased the myeloperoxidase activity (*P*<0.05; Fig 6C). The level of the superoxide dismutase activity in the T2D animals was significantly higher than in those of the CTR group (*P*<0.05). Treatment with INS or GLC and with all of the nutritional supplements significantly decreased the superoxide dismutase activity (*P*<0.05; Fig 6D).

### Bronchoalveolar lavage fluid (BALF) analysis

#### The inflammatory cell infiltration in BALF was slightly reduced by treatment with the nutritional supplements

As shown in Table 2, diabetes induction increased the total inflammatory cell numbers in the BALF. Treatment with INS or GLC increased the inflammatory cell infiltration into the BALF of the diabetic animals. The nutritional supplements, with the exception of Zn, decreased the BALF total cell numbers, but this effect did not reach statistical significance for any of the nutritional supplements groups (Table 2).

**Table 2:** Cell analysis of bronchoalveolar lavage fluid in the experimental groups. Data are least squares means and standard error of the mean.

	Treatments^2^												
Item^1^	CTR	T2D	INS	GLC	Leu	Zn	Cr	Leu-Zn	Leu-Cr	Zn-Cr	Leu-Zn-Cr	SEM	*P*-Value
Total #	4428.3^c^	5800.0^bc^	6165.0^abc^	7746.7^a^	4895.0^bc^	6354.3^ab^	4882.9^bc^	5080.0^bc^	4805.7^bc^	5270.0^bc^	4854.0^bc^	378.9	<.0001
RBC #	228.3	468.3	278.3	421.7	161.7	454.3	197.1	250.0	268.6	174.3	186.0	78.2	0.06
Non RBC #	4200.0^b^	4823.3^b^	5885.0^ab^	7325.0^a^	4733.3^b^	5900.0^ab^	4678.6^b^	4540.0^b^	4530.0^b^	5095.7^b^	4668.0^b^	383.2	<.0001
RBC/HPF	3.00^ab^	4.83^a^	2.33^ab^	3.00^ab^	2.33^ab^	4.00^ab^	1.43^b^	2.29^ab^	2.29^ab^	1.57^b^	1.61^b^	0.65	0.02
Lym/HPF	16.0^ab^	10.6^c^	12.1^bc^	17.9^a^	10.6^c^	16.0^ab^	13.6^bc^	12.3^bc^	13.4^bc^	12.7^bc^	13.0^bc^	1.01	<.0001
Epith/HPF	0.50	0.67	0.83	0.67	1.12	0.57	0.57	0.29	0.57	0.71	0.40	0.31	0.88
Macro/HPF	82.8	87.2	85.6	80.0	83.6	82.4	84.4	86.4	85.0	83.4	85.3	1.71	0.22
Goblet cell/HPF	0.15	0.49	0.33	0.51	0.5	0.00	0.29	0.29	0.14	0.43	0.23	0.21	0.80
Neut/HPF	0.66	1.33	1.17	0.83	0.8	1.29	1.14	0.71	1.00	1.00	1.00	0.31	0.91

^1^RBC = red blood cells; HPF = high power field; Lym = lymphocyte; Epith = epithelial; Macro = macrophage; Neut = neutrophil; # = number of cell

^2^CTR = control; T2D = type 2 diabetes; INS = NPH insulin; GLC = glibenclamide; Leu = leucine; Zn = zinc; Cr = chromium.

^a–c^Significant differences (*P*<0.05) among treatments are indicated by different letters.

#### Ameliorative effects of nutritional supplements on oxidative stress and protein leakage in the BALF of the diabetic rats

**Fig 7:**
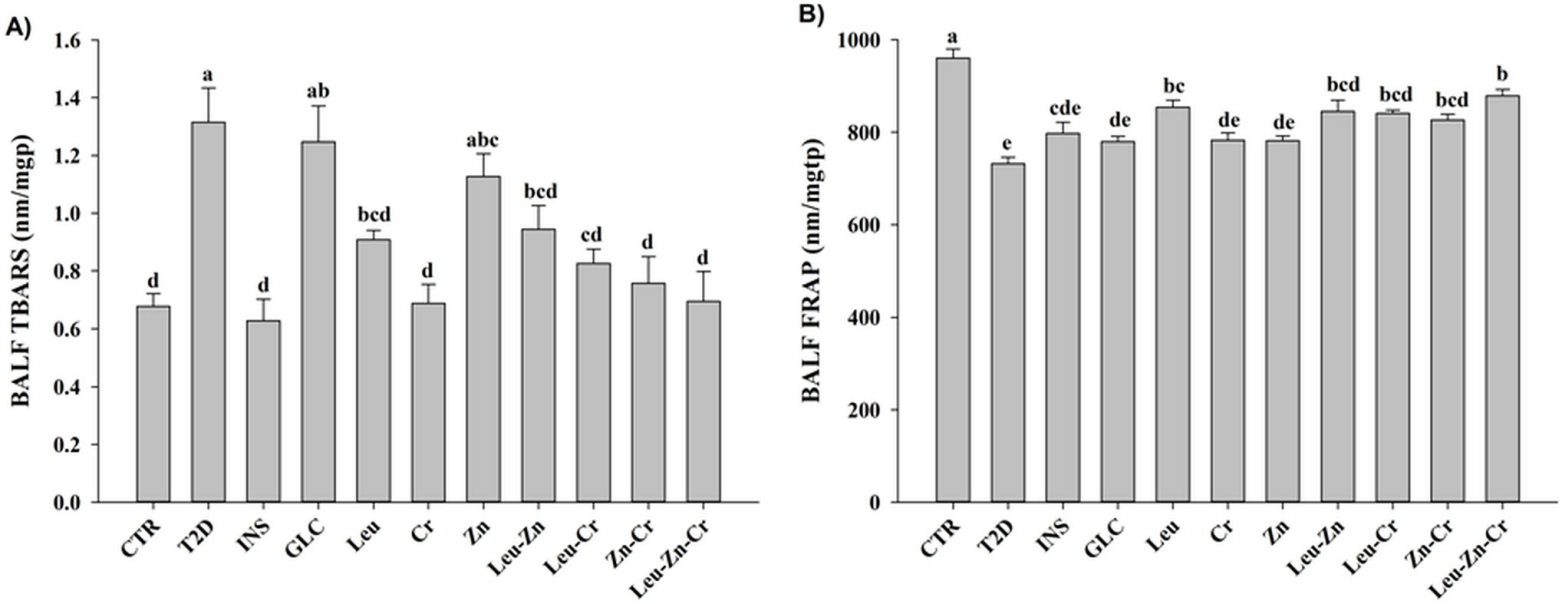
Oxidative stress in the bronchoalveolar lavage fluid (BALF) of the experimental groups. (A) TBARS (Thiobarbituric acid reactive substances) used as an index of lipid peroxidation and (B) FRAP (Ferric reducing ability of plasma) as an indicator of a total antioxidant status were measured in all of the experimental groups. Different superscript letters (a-e) indicate significant differences among the groups (*P*<0.05). CTR = control; T2D = type 2 diabetes; INS = NPH insulin; GLC = glibenclamide; Leu = leucine; Zn = zinc; Cr = chromium.

**Fig 8:**
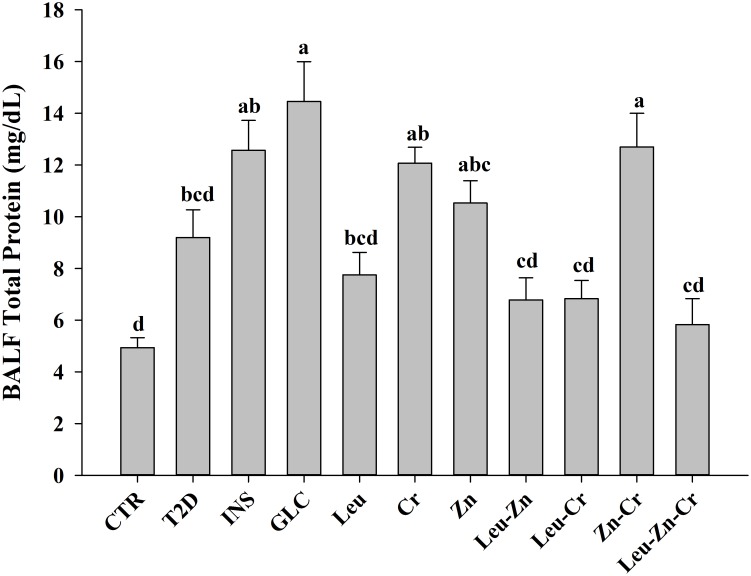
Protein leakage in the bronchoalveolar lavage fluid (BALF) of the experimental groups. The total amounts of protein were measured in the BAL fluid in all of the experimental groups. Different superscript letters (a-d) indicate significant differences among groups (*P*<0.05). CTR = control; T2D = type 2 diabetes; INS = NPH insulin; GLC = glibenclamide; Leu = leucine; Zn = zinc; Cr = chromium.

TBARS measurements (as lipid peroxidation index) and FRAP (total antioxidant status) were significantly higher and lower, respectively, in the T2D group compared with the CTR group (*P*<0.05; Fig 7A and 7B). The treatments, except GLC and Zn, significantly reduced the TBARs level in the diabetic animals (*P*<0.05; Fig 7A). Treatment with INS or GLC had no significant effect on the FRAP level in the diabetic animals. Treatment with Leu, Leu-Zn, Leu-Cr, Zn-Cr and Leu-Zn-Cr significantly increased the FRAP level in the diabetic animals (*P*<0.05; Fig 7B). As shown in Fig 8, there was an increase (*P*<0.05) in the total protein content of the BALF of the T2D animals compared with CTR subjects. Treatment with INS or GLC increased (*P*<0.05) the protein content of the BALF. Treatment with Leu, Leu-Zn, Leu-Cr or Leu-Zn-Cr slightly reduced the protein leakage in the diabetic animals, but the changes did not reach the control level (Fig 8).

### Lung biochemical analysis

#### Lung antioxidant enzyme activities improved in the diabetic animals treated with nutritional supplements

**Fig 9:**
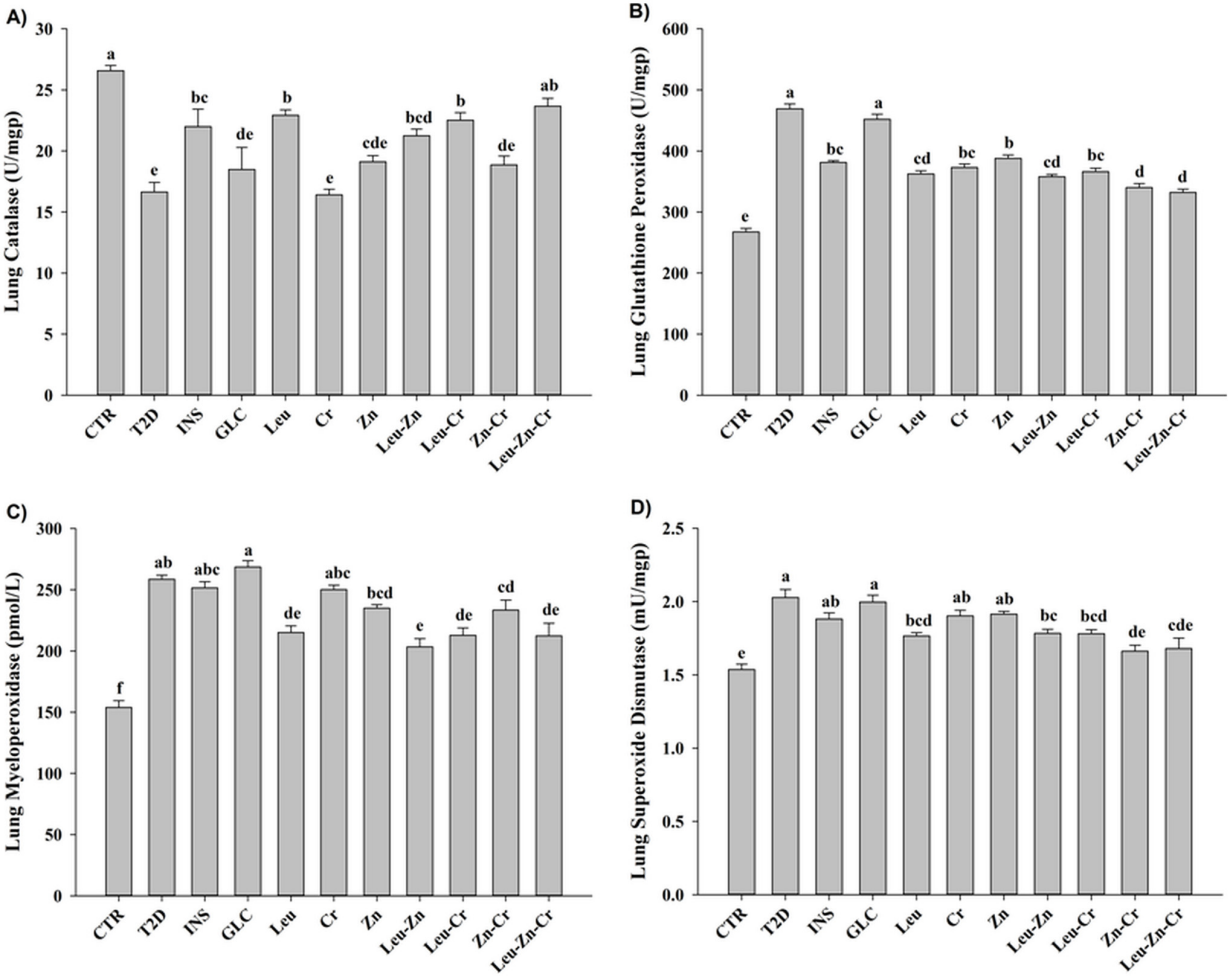
Antioxidant enzymes activities in the lungs of the experimental groups. The lung catalase (A), glutathione peroxidase (B), myeloperoxidase (C) and superoxide dismutase (D) activities were measured in all of the experimental groups. Different superscript letters (a-f) indicate significant differences among groups (*P*<0.05). CTR = control; T2D = type 2 diabetes; INS = NPH insulin; GLC = glibenclamide; Leu = leucine; Zn = zinc; Cr = chromium.

The level of catalase activity in the T2D animals was significantly lower (*P*<0.05) than that of the CTR subjects. Only treatment with INS significantly (*P*<0.05) increased the catalase activity. Treatment with Leu, Leu-Zn, Leu-Cr or Leu-Zn-Cr significantly increased the catalase activity in the lungs of the diabetic animals (*P*<0.05; Fig 9A). The glutathione peroxidase activity was significantly (*P*<0.05) increased in the T2D group compared with the CTR group. Treatment with INS or with any of the nutritional supplements or their combinations significantly decreased the glutathione peroxidase activity in the diabetic animals (*P*<0.05; Fig 9B). The myeloperoxidase activity was significantly (*P*<0.05) increased in the T2D group compared with the CTR group. Treatment with INS or GLC had no significant effect on the myeloperoxidase activity. Treatment with Leu, Leu-Zn, Leu-Cr, Zn-Cr or Leu-Zn-Cr significantly decreased the myeloperoxidase activity in the diabetic animals (*P*<0.05; Fig 9C). The level of superoxide dismutase activity in the T2D animals was significantly (*P*<0.05) higher than in the CTR animals. Treatment with INS or GLC had no significant effect on the superoxide dismutase activity in the diabetic animals. Treatment with Leu, Leu-Zn, Leu-Cr, Zn-Cr or Leu-Zn-Cr significantly decreased the superoxide dismutase activity in the diabetic animals (*P*<0.05; Fig 9D).

#### The lung oxidative stress of diabetic animals was balanced using nutritional supplements

**Fig 10:**
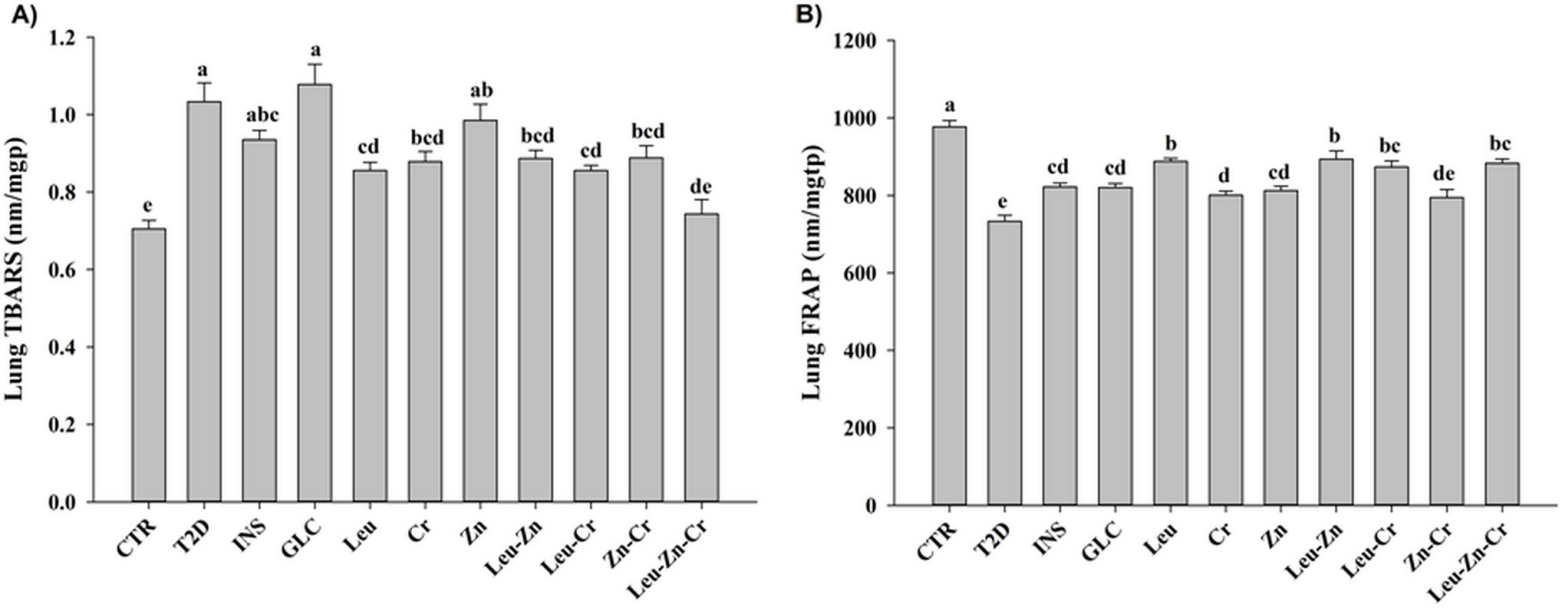
Oxidative stress in the lungs of the experimental groups. (A) TBARS (Thiobarbituric acid-reactive substances), used as an index of lipid peroxidation, and (B) FRAP (Ferric reducing ability of plasma), used to indicate the total antioxidant status, were measured in all of the experimental groups. Different superscript letters (a-c) indicate significant differences among groups (*P*<0.05).

The TBARS and FRAP measurements were significantly higher and lower, respectively, in the T2D group compared with the CTR group (*P*<0.05; F 10A and 10B). Treatment with INS or GLC had no significant effect on the TBARS level in the diabetic animals. Treatment with Leu, Leu-Zn, Leu-Cr-Zn, Zn-Cr or Leu-Zn-Cr significantly decreased the TBARS levels in the diabetic animals (*P*<0.05; Fig 10A). Except for Zn-Cr, These treatments also significantly increased the FRAP level in the diabetic animals (*P*<0.05; Fig 10B).

## Discussion

The present study showed that Leu, Zn, and Cr supplementation influences the function and histological structure of the respiratory system in a rat model of type 2 diabetes. Mounting evidence over the past few years has suggested that the lung may also be a target organ of diabetes. In the present study, we found that type 2 diabetes induction in rats resulted in the infiltration of mononuclear cells and edema in the submucosa of the trachea; hyperplasia and hypertrophy of the submucosal serous glands in the trachea; lung alveolar epithelialization; severe fibrosis around the vessels and airways; perivascular infiltration of inflammatory cells and fibrin; edema; peribronchial infiltration of inflammatory cells; hyperaemia; hypertrophy and hyperplasia of the muscles; and emphysema. In the diabetic group, the PVC, PBC, total inflammation score and Reid index significantly increased. Previous findings showed that streptozotocin-induced diabetes caused alterations in the ultrastructure of the granular pneumocytes in the inter-alveolar septae [32], the non-ciliated bronchiolar epithelial (Clara) cells [33] and the collagen and elastin in the alveolar walls [34]. Thickening of the epithelial and capillary basal laminae of the alveoli and centrilobular emphysema were also obvious findings in autopsies of diabetic subjects [35,36]. Micro-angiopathy in the capillaries of the alveolar septae and also in the alveolar and pleural arterioles has been shown in diabetic subjects [36]. Treatment with insulin efficiently ameliorated the histopathological disorders caused by diabetes in these animals. The PVC, PBC and Reid index were also significantly decreased in the INS-treated animals. In this respect, co-treatment with Zn, Cr and Leu significantly decreased the respiratory system inflammation and remodelling induced by diabetes and also normalized the histopathological changes, PVC, PBC and Reid index to levels close to the control level. Hyperglycaemia due to diabetes leads to the increased production of free radical intermediates through the following methods: increased glycolysis, intercellular activation of the sorbitol (polyol) pathway, auto-oxidation of glucose and non-enzymatic protein glycation [37,38]. It seems that the observed improvement in the histopathological changes of the respiratory system of diabetic animals treated with insulin or nutritional supplements was most likely related to the glucose-lowering effects of the treatments.

Fibrinogen, a positive acute phase protein, may be elevated in any form of inflammation. Fibrinogen levels correlate with the degree of pulmonary inflammation in lung diseases and may be involved in the increased cardiovascular risk of patients with type 2 diabetes mellitus [39,40]. In the current study, the increased serum fibrinogen and total protein concentrations observed in the T2D group were decreased in the animals treated with Leu, Zn, and Cr. In this context, the increase in the infiltration of inflammatory cells into the BALF in the diabetic rats provides direct evidence for an acute inflammatory response induced by the diabetes. The nutritional supplements used in this study decreased these features of inflammation in the diabetic animals.

In the present study, diabetes induction significantly reduced the total antioxidant status and elevated the levels of lipid peroxidation products in the serum, lung lavage and lung tissue of the T2D animals. The aetiology and pathophysiology of the biological effects of diabetes mellitus, especially with respect to cell damage, cellular degeneration, and subsequent complications are mainly attributed to oxidative stress and oxidative stress-induced apoptosis. The outcome of the long-term complications caused by oxidative stress during chronic diabetes is substantially influenced by antioxidant capacity status. In our study, the blood and lung enzymatic antioxidant activities followed similar trends in the T2D animals. The catalase activity decreased but the glutathione peroxidase, myeloperoxidase and superoxide dismutase activities increased compared to the CTR animals. Diseases, including diabetes type 1 or 2, that are accompanied by increased reactive oxygen metabolite (ROS) synthesis are associated with an initial compensatory increase in the antioxidant activities, which decrease again during prolonged oxidative stress. This effect reflects the depletion of the systemic pool of enzymes and a reduction in the antioxidant capacity [41,42]. Our findings showed that the selected nutritional supplements significantly improved the enzymatic antioxidant activities of the blood and lung to levels close to the control level. Interestingly, the trends of the effects of the treatments with the nutritional supplement on the enzymatic antioxidant activities of the blood and lung were found to be similar and showed the same beneficial trend.

It has been shown that Leu reduces oxidative and inflammatory biomarkers and increases the anti-inflammatory factor in mice and humans [43,44]. In line with our findings, it has been shown that the peroxidation of lipid molecules induced by ROS, oxidative stress and inflammation was significantly attenuated with chromium picolinate supplementation [45,46]. Zinc, an essential microelement, plays special roles in the conducting airways. The localization of the abundant labile Zn in the apical cytoplasm of the airway epithelium is controlled by specific zinc transporters [47]. Zinc affects a number of important airway proteins. Zinc increases ADAM33 (a disintegrin- and metalloproteinase-containing protein) metalloproteinase, which is important for extracellular matrix changes and airway remodelling after repeated damage and repair [48]. Zinc enhances agonist binding to the β2 adrenoceptor by cross-linking two transmembrane domains of this receptor, resulting in positive allosteric modulation and relaxation of the airway smooth muscles [49,50]. It has been shown that Zn deficiency is a positive trigger for the nuclear translocation of Nuclear Factor-kβ (NF-kβ) in rat lungs and human airway epithelial cells [51]. NF-kβ is a cytoplasmic transcription factor that, in activated cells, translocates to the nucleus and mediates expression of many pro-inflammatory cytokines [52]. Zinc has anti-inflammatory, antioxidant and pro-survival effects. Zinc deficiency leads to oxidative damage in the airways by inducing the infiltration of inflammatory cells and increasing superoxide and nitric oxide production. Zinc deficiency also exacerbates inflammation due to acute lung injury or the presence of asthma [53–55]. Taken together, all of these findings and our results suggest that Zn, Leu and Cr may be useful as potential antioxidant and anti-inflammatory dietary supplements for diabetes-induced lung inflammation and oxidative injury.

The insulinotropic effects of Leu have already been documented in previous studies [13–15]. Zinc is actively involved in insulin biosynthesis within the pancreatic β-cells and may function to modulate the insulinaemic effects in insulin-dependent tissues [16]. In addition, Cr is biologically active as a component of chromodulin, which is part of an insulin-signalling pathway and stimulates the tyrosine kinase activity of the insulin-activated insulin receptor [17,18]. Controlling hyperglycaemia by using dietary supplements, including Leu, Zn, and Cr, may reduce the hyperglycaemia-induced oxidative injuries in the respiratory tract. The most beneficial effects of the nutritional supplements on the lung functions and histological structure were observed when they were used together, which may be explained by their additive hypoglycaemic effects. Further studies may provide additional clues to the involvement of these supplements in, and the mechanisms underlying, the improved respiratory functions in the subjects with type 2 diabetes.

## Conclusions

Our study demonstrated that the supplementation of diabetic rats with Leu, Zn, and Cr, alone and in combination, was associated with significant improvements in the function and structure of respiratory system. The observed improvements may be related to anti-hyperglycaemic and antioxidant effects of the supplements. Further, supplementation of subjects with type 2 diabetes with these supplements may be an efficient method for preventing and/or treating diabetes-induced respiratory system dysfunction.

## Supporting Information

S1 Checklist NC3Rs ARRIVE Guidelines Checklist. (PDF)

S1 Dataset Raw data of study. (XLS)
